# Fine-Scale Lithogeochemical Features Influence Plant Distribution Patterns in Alpine Grasslands in the Western Alps of Italy

**DOI:** 10.3390/plants13162280

**Published:** 2024-08-16

**Authors:** Anna Cazzavillan, Renato Gerdol, Elena Marrocchino, Carmela Vaccaro, Lisa Brancaleoni

**Affiliations:** Department of Environmental and Prevention Sciences, University of Ferrara, C.so Ercole I d’Este 32, 44121 Ferrara, Italy; anna.cazzavillan@unife.it (A.C.); elena.marrocchino@unife.it (E.M.); carmela.vaccaro@unife.it (C.V.); lisa.brancaleoni@unife.it (L.B.)

**Keywords:** alpine vegetation, bedrock, calc-schist, calcareous vs. siliceous plants, geochemistry, serpentine

## Abstract

Bedrock geology is crucial in structuring alpine plant communities. Old studies mainly focused on the compositional differences between alpine plant communities on carbonate rocks and crystalline rocks, i.e., calcareous vs. siliceous vegetation. Increasing attention is being paid to bedrock types other than calcareous or siliceous ones, viz. those which have intermediate geochemical characteristics between pure calcareous and pure siliceous ones. Among these types of ‘intermediate’ bedrocks, calc-schists and serpentines are generally characterized by vegetation comprised of a mixture of basiphilous and acidophilous species. We selected several sites in alpine grasslands in the Western Italian Alps, on calc-schist and serpentine bedrocks, located at 2500 ± 100 m above sea level. X-ray fluorescence quantification of major and trace elements, combined with stereomicroscopic examination of bedrock samples with a petrographic approach, revealed a much broader range of bedrock types than recognized by inspection of geological maps. The vegetation investigated in our study was mostly composed of a set of species found more or less frequently in alpine silicicolous or calcicolous plant communities of the Alps and other European mountains. The carbonate content in the bedrock was one of the main drivers of variation in grassland vegetation, not necessarily related to soil pH. There were no distinctive species uniquely characterizing grassland vegetation on serpentines or calc-schists.

## 1. Introduction

The relationships between plant species and bedrock geology, i.e., bedrock substrate type, have long been considered one of the main factors structuring alpine plant communities. Alpine ecosystems present suitable conditions for exploring relationships between bedrock geochemistry and plant diversity, as they are characterized by ‘young’ soils such as, but not limited to, cambisols, leptosols, and regosols [1]. Furthermore, alpine ecosystems are home to approximately 20% of the Europe’s native flora [2]. The alpine altitudinal range varies geographically depending on local microclimatic conditions, yet it can be characterized by two main limits: the lower limit which coincides with the tree line, below which subalpine open woodlands are found; and the upper limit (also known as the climatic snow line) above which the snowmelt rate is lower than the annual snowfall. The grasslands represent climax, or late successional communities of the alpine elevation belt, and their maximum expression is located in the middle alpine vegetation belt. Alpine grasslands generally present a mosaic structure that reflects a strong heterogeneity of microhabitats due to both macro- and micro-topography, and their interaction with environmental factors such as incident radiation, wind, water availability, and soil nutrient content [3,4,5]. Seminal studies conducted in the European Alps have mainly focused on the compositional differences between alpine plant communities on calcareous rocks and siliceous rocks [6,7,8]. The strong differences between calcareous and siliceous high-altitude vegetation are associated with various adaptations of plant species to these two bedrock types; i.e., the so-called calcicole vs. calcifuge strategies, with calcicole species limited to calcareous bedrocks, calcifuge species limited to siliceous bedrocks, and a set of indifferent species growing together on the two bedrock types [9]. Different ecophysiological adaptations of calcicole vs. calcifuge plants have been documented. In particular, calcicole species are adapted to low availability of elements, especially Fe and Mn, and to a lesser extent Zn, Cu and B, as well as to high levels of Ca with adverse effects on plant K uptake in alkaline calcareous soils. On the other hand, calcifuge species are adapted to low Ca, Mg, Mo, and P availability, but also to the potentially toxic effects caused by the high solubility of Al, Mn, and Fe in acidic siliceous soils [10,11,12,13,14,15,16].

In recent years, bedrock types other than pure calcareous or siliceous bedrocks are receiving increasingly more attention. Among these, mafic-ultramafic rocks (commonly called serpentines) and calcite-containing schists (commonly called calc-schists) present geochemical features intermediate between those of pure calcareous and pure siliceous bedrocks. In particular, soils derived from serpentines and calc-schists usually fall within a narrower pH range than strongly acidic soils originating from siliceous rocks on the one hand and alkaline soils originating from calcareous rocks on the other. As a result, vegetation on serpentines and calc-schists is often composed of a mixture of calcicole and calcifuge species without the clear separation that usually occurs between pure calcareous or siliceous bedrocks. Serpentines and, to a lesser extent, calc-schists, represent rather broad petrologic types. Serpentines comprise a large set of rocks with varying degrees of metamorphism that can differ considerably from each other in terms of mineralogic composition and original protolith [17,18]. Calc-schists can also present notable variations regarding the original protolith and, above all, calcite content [19,20]. These petrographic variations can in turn influence the soil chemistry and vegetation composition of plant communities settled on serpentines and calc-schists.

The western sector of the Alps, henceforth called the Western Alps, presents by far the greatest petrographic variety across the entire Alpine chain. Bedrock types in the Western Alps range from alkaline calcareous rocks such as limestone and dolomite to acidic siliceous rocks such as granite and quartzite [21]. Much of the Western Alps are geologically composed of bedrock types that originated from the ancient Jurassic Tethyan oceanic basement [22,23]. They are predominantly made of serpentines and calc-schists which are found in close association with each other in different areas from the southern Cottian Alps to the northern Pennine Alps [24]. This region is therefore particularly suitable for exploring relationships between alpine vegetation and bedrock geochemistry. The objective of this paper was to analyze relationships between vegetation and bedrock geochemistry in order to disentangle the complex interactions that drive patterns of plant biodiversity in alpine grasslands ecosystems on serpentines and calc-schists.

## 2. Results

### 2.1. Bedrock Typification Based on Geological Maps and Stereomicrosopic Observations

According to geological maps [24], the serpentines s.l., otherwise called ophiolites s.l., represent an extremely heterogenous group, including prasinites, amphibolites, eclogites, jadeites, serpentines, eclogitic mica-schists, talc-schists, actinolitic chlorite-schists, grenatiferous chlorite schists, and pyroxenites. The calc-schists s.l. generally included various types of lime-rich bedrocks. However, they often presented intercalations, especially of mica-schist layers (e.g., white mica, micaceous gray limestones and others), fine gneisses, quartzites, and phyllites (Supplementary Table S1) due to their schistosity. This binary classification proved effective during preliminary field recognition, but microscopical and geochemical analyses proved otherwise. Although the serpentines s.l. were easily recognizable in the field, and clearly differentiated from calc-schists s.l., microscopic observations revealed much higher heterogeneity, suggesting further possible groupings within the two main lithological groups (Supplementary Table S2).

### 2.2. Bedrock Typification Based on Geochemistry

**Figure 1:**
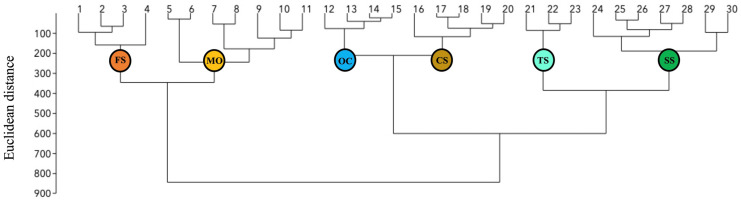
Classification dendrogram of normalized geochemical variables. The circles indicate six clusters, identified at Euclidean distance of about 300, corresponding to the lithological groups (FS: relatively felsic schists; MO: meta-ophiolites; OC: meta-ophicalcites; CS: calc-schists s.s.; TS: talc-schists; SS: serpentine-schists).

Two main clusters were defined in the classification dendrogram at a Euclidean distance of approximately 800 (Figure 1). The first cluster included plots 1–11, where the bedrock was rich in sialic components, such as SiO_2_, Al_2_O_3_ and K_2_O, together with many elements often associated with metamorphic fluids [25], namely Ba, Ce, Ga, Hf, Nb, Rb, Th, Zn, and Zr (Table 1). The second cluster included plots 12–30 with a low K_2_O content, and a medium-low SiO_2_ content, but with a very heterogenous geochemical composition (Figure 1; Table 1). From a petrological point of view, plots 1–11 formed a heterogenous group, with meta-gabbros, mica-schists (both calcareous and non-calcareous), and chlorite-schists. Protoliths of these lithological types are usually considered as ’alkaline’, especially when considering minerals derived from mafic rocks, such as chlorite, and mafic rocks themselves, such as gabbros. Nonetheless these plots had a higher silica content than mafic rocks and other alkaline rocks.

Six clusters were defined in the dendrogram at Euclidean distance of approximately 300 (Figure 1). These clusters were considered as fine-scale lithological groups in our sample set. Briefly, the six groups were defined as follows: moderately felsic schists (FS), meta-ophiolites (MO), meta-ophicalcites (OC), calc-schists s.s. (CS), talc-schists (TS), and serpentine-schists (SS). The six lithological groups differed significantly from each other in terms of all variables except La (Table 1). The three variables with greatest significance among groups were, in decreasing order, Ni, MgO, and CaCO_3_ (Table 1). From a geochemical point of view, the six lithological groups were characterized as follows.

- FS did not contain CaCO_3_ and had low contents of Ni and MgO as well as of other mafic indicators such as Co and Cr. However, FS was quite rich in Fe_2_O_3_, another mafic indicator. FS showed the highest contents of SiO_2_, Al_2_O_3_, Na_2_O and P_2_O_5_, Ba, Ce, Ga, Hf, La, Nd, Th, and Zr (Table 1). The rather high Na_2_O content was presumably linked to Na-rich plagioclase feldspar, a discriminating mineral in intermediate rocks. From a petrological point of view, the FS group was heterogeneous and included both meta-gabbros and non-calcareous mica-schists.
- MO had an intermediate CaCO_3_ content but had discrepancies in the concentrations of mafic indicators, with intermediate contents of MgO, Fe_2_O_3_, Co, and Cr but high concentrations of Ni and Cu. Furthermore, MO presented high concentrations of MnO, K_2_O, Ba, Ga, Nb, Nd, Pb, Rb, Th, and Zn (Table 1). Petrologically, MO also was heterogeneous and included chlorite schists together with calcareous and non-calcareous mica-schists.
- OC was a hybrid lithological group with intermediate characteristics between ophiolites s.l. and calc-schists s.l. This was due to the medium-high concentrations of CaCO_3_, CaO, MgO, Ni, and Sr. OC also had a high Pb content and was La-free (Table 1).
- CS had the highest contents of CaCO_3_, CaO, and Sr and lower concentrations of Fe_2_O_3_, MgO, Cr, Ni, Sc, and Zn than almost all other lithological groups (Table 1).
- TS had extremely high contents of mafic indicators such as MgO, Co, Cr, and Ni. Furthermore, TS did not contain CaCO_3_, K_2_O, Ga, Hf, La, and Nd (Table 1).
- SS had a particular geochemical composition. Similar to TS, this group was rich in MgO, Ni, Co, Cr, and Sc and had high concentrations of TiO_2_, Fe_2_O_3_, MnO, Na_2_O, P_2_O_5_, V, and Y as well (Table 1).

### 2.3. Direct Solar Radiation (DSR) and Soil Chemistry

**Figure 2:**
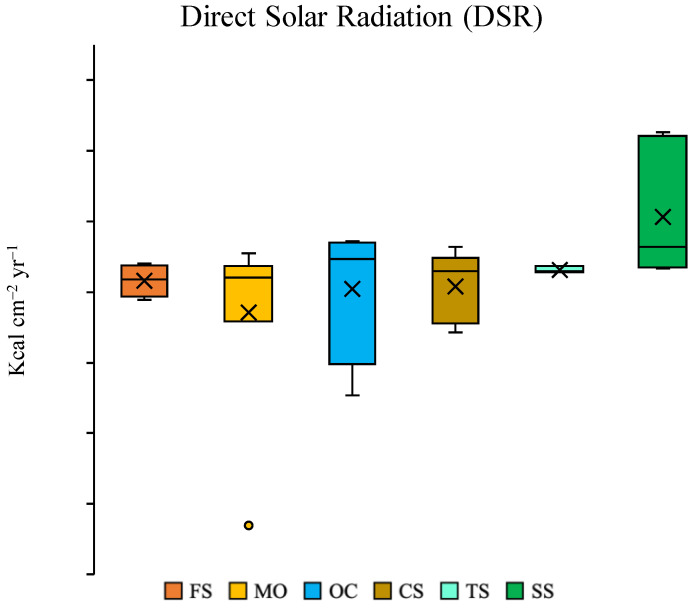
Box plots of direct solar radiation (DSR) in the six lithological groups. The box represents the 25–75 percent quartiles, the horizontal line is the median, the whisker indicates minimum and maximum values, the cross indicates the mean values, the circle indicates the outlier. FS: moderately felsic schists; MO: meta-ophiolites; OC: meta-ophicalcites; CS: calc-schists s.s.; TS: talc-schists; SS: serpentine-schists (SS).

Although there were no overall differences among lithological types in terms of DSR (F_5,24_ = 1.69; *p* = 0.17), the mean DSR was significantly different (F_1,12_ = 4.94; *p* < 0.05) between the highest value in SS and the lowest value in MO (Figure 2). The soil VWC also differed significantly (F_1,12_ = 18.74; *p* < 0.001) between these two lithological types with a higher value in MO (11.3 ± 1.3%) than in SS (5.1 ± 0.5%).

**Figure 3:**
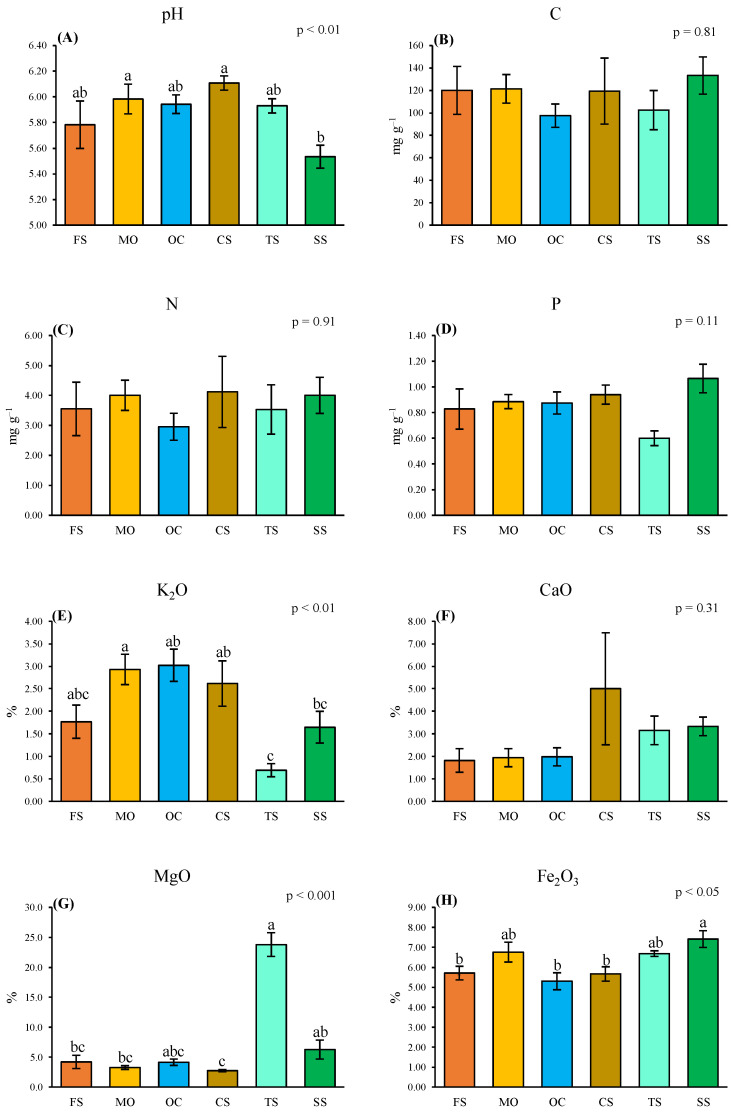
Mean (±1 SE) values of pH (**A**), total concentrations of carbon (**B**), nitrogen (**C**), phosphorus (**D**), potassium (**E**), calcium (**F**), magnesium (**G**), and iron (**H**) in the soil of the six lithological groups, with the relative *p* levels obtained by one-way ANOVAs. For each variable the means followed by the same letter do not differ significantly (*p* < 0.05) based on Dunn’s post hoc tests. FS: moderately felsic schists; MO: meta-ophiolites; OC: meta-ophicalcites; CS: calc-schists s.s.; TS: talc-schists; SS: serpentine-schists (SS).

The soil pH differed among the lithological groups, with the SS soils being significantly more acidic than the CS and MO soils (Figure 3A). The K concentration was highest in MO, OC, and Cs; intermediate in FS and SS; and lowest in TS (Figure 3E). The Mg concentration was distinctly higher in TS (Figure 3G) and the Fe concentration was slightly higher in SS than in the other lithological groups (Figure 3H). The concentrations of C, N, P, and Ca were more or less the same in all lithological groups (Figure 3B–D,F).

### 2.4. Vegetation and Its Relationships with the Lithological Groups and Bedrock Chemistry

In total, 137 species were recorded, of which 76 were present in 3 or more plots (Table 2). The other 61 species were considered ‘occasional species’ and are listed in Supplementary Table S3. On the basis of the IndVal index, 38 species were identified, among which 9 were highly indicative (*p* < 0.01; Table 2). The indicator species are listed below.

- FS indicator species: *Bartsia alpina*, *Mutellina adonidifolia*, *Phyteuma hemisphaericum*, *Scorzoneroides helvetica*, and *Veronica bellidioides*.
- MO indicator species: *Achillea nana*, *Pedicularis kerneri*, *Salix retusa*, *Anthoxanthum nipponicum*, *Festuca nigricans*, and *Bistorta vivipara*.
- OC indicator species: *Helianthemum nummularium* subsp. *grandiflorum*, and *Silene acaulis*.
- CS indicator species: *Helianthemum oelandicum* subsp. *alpestre*, *Botrychium lunaria*, *Draba aizoides* subsp. *aizoides*, *Erigeron uniflorus*, *Pedicularis rosea* subsp. *allionii*, *Phyteuma globulariifolium* subsp. *pedemontanum*, *Plantago alpina*, *Poa alpina*, *Saxifraga oppositifolia* subsp. *oppositifolia*, and *Jacobaea incana*.
- TS indicator species: *Luzula lutea* subsp. *lutea*, *Potentilla crantzii* subsp. *crantzii*, *Thymus praecox* subsp. *polytrichus*, *Agrostis rupestris* subsp. *rupestris*, *Dianthus furcatus*, *Festuca rubra*, *Galium anisophyllon*, and *Oreojuncus trifidus*.
- SS indicator species: *Juncus jacquinii*, *Alchemilla vulgaris*, *Festuca halleri*, *Festuca scabriculmis* subsp. *luedii*, *Nardus stricta*, *Pulsatilla alpina* subsp. *apiifolia*, and *Sempervivum montanum*.

**Figure 4:**
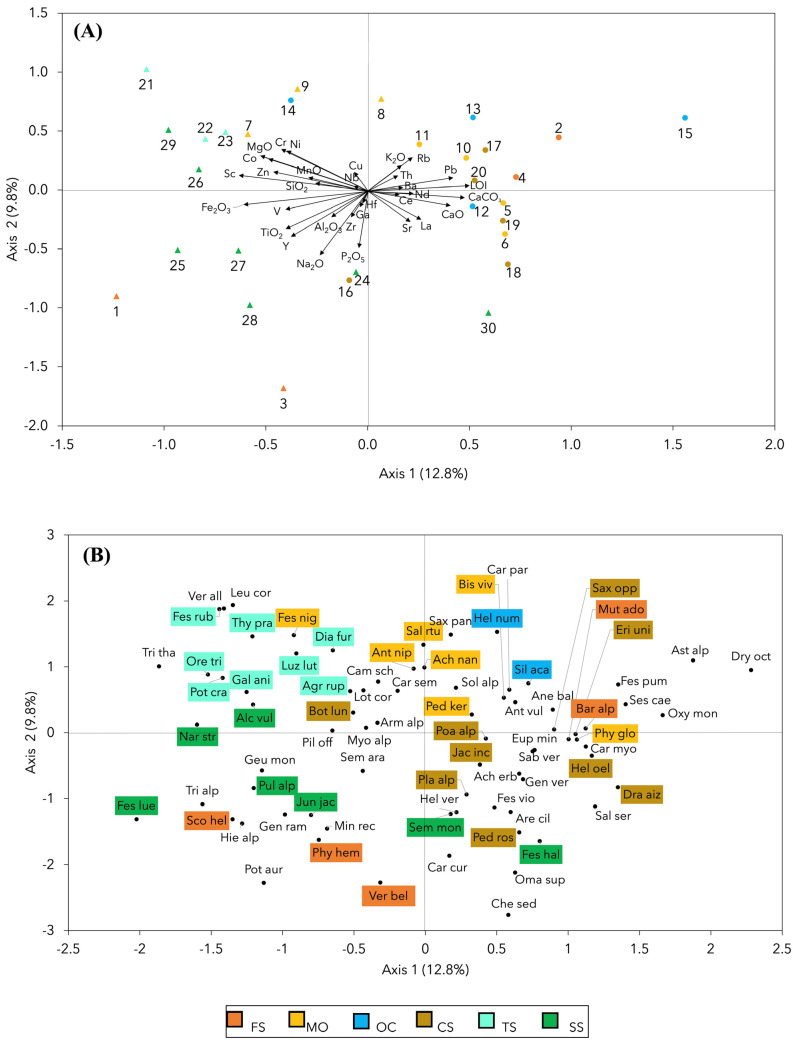
Reciprocal ordination of vegetation and bedrock chemistry along the first two Canonical Correspondence Analysis (CCA) axes (percentage of variance accounted for each axis in parentheses). Top panel (**A**): biplot of 30 sampling plots and 32 geochemical variables. The plots are grouped by lithological groups with different colors as in the legend (FS: moderately felsic schists; MO: meta-ophiolites; OC: meta-ophicalcites; CS: calc-schists s.s.; TS: talc-schists; SS: serpentine-schists). Triangles indicate plots corresponding to serpentines s.l.; circles indicate plots corresponding to calc-schists s.l. based on binary classification in the field. Bottom panel (**B**): plot of 76 species. The indicator species of the six lithological groups are highlighted by colors as in the legend. Species abbreviations as in Table 2.

The first two CCA axes (Figure 4A,B) collectively accounted for 22.6% of the total variance. The ordination of the plots along the first CCA axis was mainly associated with increasing concentrations of CaO, CaCO_3_, L.O.I. and Pb towards the right end of the biplot. In contrast, the Fe_2_O_3_ vector had an opposite orientation, directed towards the left end of the biplot (Figure 4A). The ordination of species (Figure 4B) along the first CCA axis generally reflected a soil pH gradient, with calcifuge species having negative scores and calcicole species positive scores on the first CCA axis. However, species ordination along the first CCA axis only partly reflected associations between species and lithological groups. Notably, all calcicole species at the right end of the gradient (*Dryas octopetala* subsp. *octopetala*, *Aster alpinus* subsp. *alpinus*, *Festuca pumila*, *Oxytropis montana*, and *Sesleria caerulea*) did not represent indicator species for any lithological group. However, most CS indicator species represented moderately calcicole species with positive scores on the first CCA axis. This was especially the case for *Draba aizoides* subsp. *aizoides*, *Helianthemum oelandicum* subsp. *alpestre*, *Erigeron uniflorus*, and *Saxifraga oppositifolia* subsp. *oppositifolia*. Other CS indicator species presented only weakly calcicole characteristics (*Pedicularis rosea* subsp. *allionii*, *Phyteuma globulariifolium* subsp. *pedemontanum,* and *Poa alpina*) or even more or less strongly calcifuge characteristics (*Plantago alpina*, *Jacobaea incana,* and *Botrychium lunaria*). Consistently, these species had less positive or even slightly negative scores on the first CCA axis (Figure 4B). Further mutual relationships of plant species and plots in relation to bedrock chemistry were revealed by the combined scores on the two CCA axes. The TS indicator species (*Agrostis rupestris* subsp. *rupestris*, *Dianthus furcatus*, *Festuca rubra*, *Galium anisophyllon*, *Oreojuncus trifidus*, *Luzula lutea* subsp. *lutea*, *Potentilla crantzii* subsp. *crantzii*, and *Thymus praecox* subsp. *polytrichus*) and all TS plots were located in the upper left sector of the diagram, i.e., the side towards which the vectors of Ni, Cr, Co, Zn, Sc, and MgO were oriented (Figure 4A,B). The MO plots were distributed in a wide range of scores on the first CCA axis with an overall poor association with the vectors of the bedrock chemistry variables, except Rb and to a lesser extent K_2_O (Figure 4A). The MO indicator species (*Achillea nana*, *Anthoxanthum nipponicum*, *Festuca nigricans*, *Pedicularis kerneri*, *Bistorta vivipara*, and *Salix retusa*) were also distributed in a wide range in the upper sector of the diagram (Figure 4B). The SS plots were located in the lower left sector of the diagram in close association with the Fe_2_O_3_, V, TiO_2_ and Y vectors, as well as with most SS indicator species (*Alchemilla vulgaris*, *Festuca scabriculmis* subsp. *luedii*, *Juncus jacquinii*, *Nardus stricta*, *Pulsatilla alpina* subsp. *apiifolia*, and *Sempervivum montanum*) with the exception of *Festuca halleri* which was located in the lower right sector of the diagram (Figure 4A,B). The OC plots were mostly located in the upper right sector of the diagram which reflected their quite high CaO and CaCO_3_ contents and also the high L.O.I., although all were slightly lower than those of the CS (Table 1). The two OC indicator species (*Helianthemum nummularium* subsp. *grandiflorum*, and *Silene acaulis*) were also located in the upper right sector of the diagram (Figure 4A,B). The reciprocal ordination of the FS plots and their indicator species reflected the heterogeneity of this lithological group in terms of bedrock chemistry, soil pH, and species composition. In fact, plots 1 and 3 were located in the lower left sector of the diagram, i.e., the side towards which the vectors of SiO_2_, Al_2_O_3_, TiO_2_, Na_2_O, and P_2_O_5_ were oriented (Figure 4A). These two FS plots were characterized by acidic pH and, consistently, by the presence of three calcifuge indicator species (*Phyteuma hemisphaericum*, *Scorzoneroides helvetica*, and *Veronica bellidioides*), also located in the lower left sector of the diagram (Table 2 and Figure 4B). On the contrary, plots 2 and 4 were located in the upper right sector of the diagram in association with the Ce and La vectors. These two plots had higher pH and were characterized by the indicator species *Bartsia alpina* and *Mutellina adonidifolia*, which are slightly calcicole or indifferent (Table 2 and Figure 4B).

## 3. Discussion

The vegetation of the grasslands analyzed in our study was not distinctive of calc-schists s.l. or serpentines s.l. Indeed, most of the species recorded in our plots are considered characteristic of alpine grasslands which comprise a range of plant communities across mountain ranges and arctic regions in Europe. The majority of these species characterize the alpine and subalpine silicicolous or calcicolous communities of the mountain ranges in the nemoral zone of Europe (*Caricetalia curvulae* and *Seslerietalia caeruleae*, respectively [26]). A minority of the remaining species characterize alpine habitats other than true grasslands [26]: relict summit graminoid tundra in the alpine and subnival belts (*Oxytropido-Elynetalia*); subnival and alpine lime-rich shale screes and frozen slopes of the Alps and the Pyrenees of the nemoral mountain ranges (*Drabetalia hoppeanae*); snow-beds on stabilized calcareous screes of the arctic zone and the alpine and subnival belts of European mountains (*Arabidetalia caeruleae*); arctic and alpine subnival snow-beds at high altitudes of the mountain ranges of Eurasia and the Arctic Ocean islands (*Salicetalia herbaceae*). About one-fifth of the species are characteristic of other syntaxa, not specifically related to alpine habitats (Table 2). This confirms the results of previous studies reporting the lack of distinctive vegetation types in alpine grasslands on calc-schist s.l. or serpentine s.l. bedrocks in the Western Alps [27,28]. The only distinctive plant community in the serpentine alpine habitats of the Western Alps is the *Caricetum fimbriatae*. However, this community is generally located in the upper parts of stabilized screes and especially in rock crevices where the character species *Carex fimbriata* is often associated with the chasmophyte *Cardamine plumieri* [29]. These habitats were outside the alpine grasslands sampled in our study. Consistently, *Carex fimbriata* was never recorded in our plots. There was no clear separation between calcifuge and calcicole species on calc-schist s.l. and serpentine s.l. bedrocks. Calcifuge species, characteristic of *Caricetalia curvulae*, were overall more frequent in the more acidic serpentine plots. However, among the frequent species (i.e., occurring in at least 30% of the plots) of *Caricetalia curvulae*, only *Oreojuncus trifidus* and *Trifolium alpinum* were present exclusively, or almost exclusively, in the serpentine s.l. plots. The typical species of *Caricetalia curvulae*, such as *Carex curvula* subsp. *curvula* and *Helictotrichon versicolor* subsp. *versicolor*, were even recorded with equal frequency in the two main bedrock types. A slightly higher number of calcicole species characteristic of *Seslerietalia caeruleae* (*Helianthemum oelandicum* subsp. *alpestre*, *Sesleria caerulea*, *Anthyllis vulneraria* subsp. *alpicola,* and *Oxytropis montana*) or *Oxytropido-Elynetalia* (*Dryas octopetala* subsp. *octopetala* and *Carex myosuroides*) were much more frequent in, or entirely excusive of, the calc-schist s.l. plots. Also in this case, none of these species characterize the vegetation of calc-schists s.l. or serpentines s.l. since they are widespread in alpine plant communities on siliceous bedrocks such as granite and gneiss, especially *Caricetum curvulae*, or carbonate rocks, especially *Seslerio-Caricetum sempervirentis*, *Caricetum firmae*, and *Elynetum myosuroidis*, respectively [30].

The aim of our study was precisely to investigate whether and to what extent a detailed geochemical and petrographic analysis, beyond the dichotomous distinction between calc-schists s.l. and serpentines s.l., could be effective for a fine characterization of alpine grasslands based on species composition. The overall geological complexity of the Western Alps was partially reflected in our lithological samples, which presented considerable geochemical heterogeneity including both continental and ophiolitic nappes, known as the Penninic domain [31]. The so-called ophiolitic basement nappes exhibit a high-grade metamorphism and are overlain by a relatively wide array of metasediments ranging from calc-schists to marbles, quartzites, black schists, shales, metabasites, metaophicalcites, and radiolarian cherts [32,33]. In the geographic context of the Penninic domain of the Western Alps, three macro-categories of ophiolites were identified [34]: (1) ultramafic peridotitic rocks from the asthenosphere, mostly serpentinized by hydrothermal metamorphism into serpentinites; (2) mafic rocks such as metagabbros and metabasalts, both originating from the ocean crust and presenting inclusions of micas and feldspar; (3) pelagic metasediments, both siliceous and calcareous (e.g., calc-schists, ophicalcites and serpentine breccias with calcareous cement). Therefore, the definition of ophiolites lacks geochemical homogeneity due to the substantial differences between crustal and mantle rocks, which impose different constraints on the vegetation [35]. The two lithological groups entirely made up of plots assigned to serpentines s.l., viz. TS and SS, were both quite well characterized in terms of species composition. In particular, TS was characterized by *Galium anisophyllon*, a species of metallicolous vegetation (*Galio anisophylli-Minuartion vernae*) from central Europe [36]. *Sabulina verna* (=*Minuartia verna*). Another character species of *Galio anisophylli-Minuartion vernae*, was also present in one of the TS plots but it was absolutely not an indicator species of TS because it was also recorded with equal frequency in OC and CS. Furthermore, TS was also characterized by *Luzula lutea* subsp. *lutea,* considered as a serpentine indicator in the Western Alps [37]. These vegetation characteristics are consistent with the bedrock geochemistry in TS, which had high concentrations of MgO and heavy metals, especially Co, Cr, and Ni. The latter reflect the high heavy metal contents detected in serpentine soils of different regions across the world [38,39,40,41,42]. Although none of these three species are limited to serpentine grasslands, all can behave as heavy metal accumulators [43,44] which have evolved physiological mechanisms to tolerate heavy metal toxicity [45,46,47]. The soil chemistry reflected the geochemical characteristics of the TS bedrock. Although our study did not aim to assess the availability of individual elements to plants, which would require analysis of extractable elements [38], the total concentrations of major nutrients in the TS soils provided evidence of P and especially K limitation as often observed in serpentine soils [48]. Some of the TS indicator species were also present in SS, although with lower frequencies, in agreement with the lower contents of MgO and heavy metal in the SS bedrock compared to that of TS. On the other hand, the SS indicator species, especially *Festuca scabriculmis* subsp. *luedii*, are typical of alpine grasslands developed on steep slopes with stony and dry soils, as evidenced by the high DSR and low soil VWC in the SS plots. This vegetation resembled that of alpine grasslands on steep arid south-facing slopes developed on different types of siliceous bedrocks, such as granite, gneiss, porphyry, and others, in several sectors of the Alps. The vegetation of these grasslands is characterized by taxonomically closely related calcifuge taxa of the *Festuca varia* group [49,50]. This suggests that other factors in addition to bedrock chemistry, i.e., solar radiation input and soil water content, could explain some degree of variation in the species composition of the alpine grasslands considered in our study.

The two lithological groups entirely composed of plots assigned to calc-schists s.l., viz. OC and CS, were quite well characterized in terms of species composition: *Carex myosuroides*, *Carex curvula* subsp. *curvula*, and *Helianthemum oelandicum* subsp. *alpestre* (the latter especially in CS) were among the most frequent species in the vegetation of these two lithological groups. All of them are dominant in alpine grasslands on calc-schists in the Vanoise Massif of the French Alps, near the Italian border [51]. In the calc-schist grasslands of the Vanoise, *Carex curvula* subsp. *rosae* has generally been recorded, while in our plots only *Carex curvula* subsp. *curvula* was present. Among the CS indicator species, *Saxifraga oppositifolia* subsp. *oppositifolia* and *Poa alpina* were of great importance because they characterize the vegetation of cryoturbated alpine habitats on calc-schist bedrock in the Western Pennine Alps in Switzerland [52]. Furthermore, the set of the CS indicators included three calcicole species which represent diagnostic species of stabilized alpine debris on calc-schist bedrock (order *Drabetalia hoppeanae*): *Phyteuma globulariifolium* subsp. *pedemontanum*, *Erigeron uniflorus*, and *Draba aizoides* subsp. *aizoides* [53]. Some of the CS indicator species were also occasionally found in OC, but the most distinctive indicator species for OC was *Silene acaulis*, a cushion species widespread throughout the Northern Hemisphere on different types of bedrocks [54,55]. The vegetation characteristics described above were consistent with the distinctive geochemical signature of the OC and CS bedrock, characterized by high CaO and CaCO_3_ contents with peak values in CS. This supports the results of other studies that have focused on carbonate content as the main driver of variation in alpine vegetation [56,57]. FS and MO had the common characteristic of sialic rocks, consisting of high contents of SiO_2_, Al_2_O_3,_ and K_2_O. Apart from this, both lithological groups were petrologically heterogeneous to the point of including plots classified both as serpentines s.l. and calc-schists s.l. This heterogeneity was reflected by the vegetation characteristics of FS and MO. The FS indicators consisted mainly of calcifuge species characteristic of *Caricetalia curvulae*: *Phyteuma hemisphaericum*, *Scorzoneroides helvetica,* and *Veronica bellidioides*. The MO indicators were ecologically more heterogeneous, with calcifuge species typical of *Caricetalia curvulae* (*Pedicularis kerneri*) associated with calcicole species typical of *Seslerietalia caeruleae* (*Festuca nigricans*) and moderately calcicole species typical of stabilized calc-schist screes of *Drabetalia hoppeanae* (*Achillea nana*). This is consistent with the bedrock chemistry in MO, presenting higher CaO and CaCO_3_ contents compared to FS. This, once again, underlines the important role that carbonate content plays in structuring alpine vegetation, not necessarily correlated with soil pH [56]. Interestingly, the set of MO indicators also included *Salix retusa*, a dwarf-willow characterizing snow-bed vegetation on carbonate soils (*Arabidetalia caeruleae*; [58]). An additional MO indicator, which was present in all MO plots, was *Bistorta vivipara*. *Bistorta vivipara* is a widespread species found in several alpine plant communities but is particularly frequent in snow-beds where it is enhanced by meltwater that saturates the soil at the beginning of the growing season [59]. Similar to what was observed for SS, this suggests that environmental factors unrelated to bedrock chemistry, in this case snow cover duration, which is in turn related to lower solar radiation input and higher soil VWC, were responsible for a degree of variation in the investigated alpine grasslands.

## 4. Materials and Methods

### 4.1. Study Area, Sampling Design, and Data Collection

**Figure 5:**
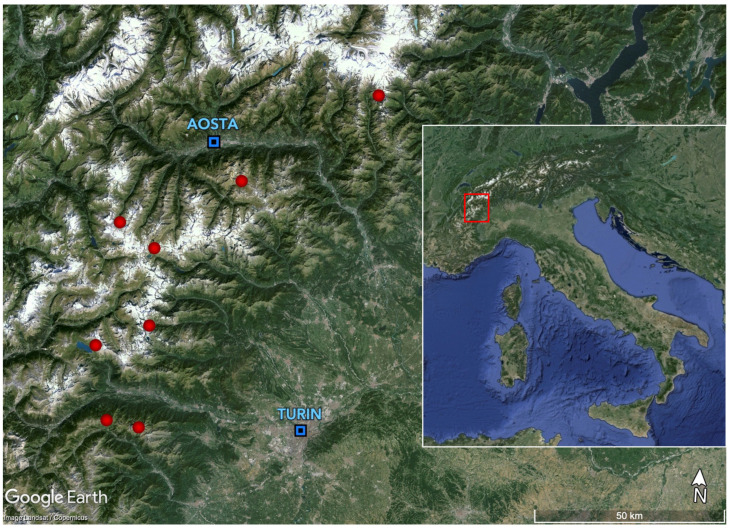
The study area. The red dots indicate the sampling sites.

Our goal was to sample a region large enough to cover the widest possible range of geographic variation in the petrographic composition of the two bedrock types examined. With this objective in mind, we selected eight sites in alpine grasslands in a region ranging between the northern Cottian Alps, the whole Graian Alps, and the middle Pennine Alps (Figure 5). These sites were chosen after a careful inspection of geological maps [24] which allowed us to select a series of sites where rather vast outcrops of serpentines, calc-schists, or both bedrock types were present. At each of the 8 sites we selected 3 to 6 1 × 1 m square plots, for a total of 30 plots. The small size of the sampling plots was justified by the need to sample petrographically homogeneous plots because it is known that rock and soil chemistry can present small-scale variation in relation to microtopography [60]. The choice of the sampling plots was based on a careful visual inspection of the sites. All plots were located at 2500 ± 100 m above sea level, on flat or moderately inclined terrain (maximum slope inclination of 35°), thus limiting the sampling area to the mid-alpine vegetation belt, i.e., where the alpine grasslands thrive and reach their late successional stages. At each site, the location of the plots covered the whole range of aspects where appropriate areas were found, in most cases all four cardinal points. Regarding vegetation structure, all plots were located in areas where vegetation covered at least 50% of the ground but vegetation cover generally was >60%. Only areas covered by low stature vegetation were considered, i.e., with a maximum total shrub cover < 10%, except for dwarf willows (*Salix herbacea*, *S. reticulata*, *S. retusa*, *S. serpillifolia*) and alpine azalea (*Kalmia procumbens*). The nomenclature of the species follows the Portal to the Flora of Italy [61].

The field sampling was carried out during the period 12–27 July 2022. The cover of all vascular species was estimated visually at each plot using the following categorical scale: 1, cover 1–10%; 2, cover 11–20%; 3, cover 21–30%; 4, cover 31–40%; 5, cover 41–50%; 6, cover 51–60%; 7, cover 61–70%; 8, cover 71–80%; 9, cover: 81–90%; 10, cover: 91–100%. Geographic coordinates, elevation, aspect, and slope angle were determined at each plot by a GPS and a compass. At some plots we also determined soil volumetric water content (VWC) using a FieldScout time domain reflectometer TDR 100 Soil Moisture Meter (Spectrum Technologies Inc., Aurora, IL, USA). At each plot, a rock sample was collected from natural outcrops and about 100 g of the top soil was collected as well. All plots had very shallow soils. Thus, soil sampling was carried out by removing the top 1 cm layer and then collecting the soil to a maximum depth of 5 cm, which is the depth to which almost all plants root. The soil sample for a given plot consisted of composite material collected from at least five points spread over the 1 × 1 m^2^.

### 4.2. Bedrock and Soil Analyses

The bedrock analyses were carried out at the Department of Physics and Earth Sciences of Ferrara University. The rock samples were examined under a stereomicroscope to obtain a detailed description of the mineral structure. A rock subsample was pulverized with an agate pestle and three aliquots were assigned to different analyses: the first aliquot was used for calcimetric analyses that evaluated the CaCO_3_ content by the gas volumetric method; the second aliquot was used to calculate L.O.I (loss on ignition); the third aliquot was incorporated into a tablet prepared with a boric acid support in order to determine concentrations of major and trace elements by X-ray fluorescence (X.R.F.) analysis using a wavelength-dispersive ARL Advant’XP X-ray fluorescence spectrometer (Thermo Fisher Scientific, Waltham, MA, USA). The main elements were expressed as percentage of oxide weight (SiO_2_, TiO_2_, Al_2_O_3_, Fe_2_O_3_, MnO, MgO, CaO, Na_2_O, K_2_O, P_2_O_5_). Concentrations of trace elements (Ba, Ce, Co, Cr, Cu, Ga, Hf, La, Nb, Nd, Ni, Pb, Rb, Sc, Sr, Th, V, Y, Zn and Zr) were expressed as mg kg^−1^. The entire matrix correction procedure and the intensities were elaborated according to Lachance and Traill [62]. The accuracy of the instrumentation was estimated on the basis of the results obtained from international standards of geological samples, and the precision was expressed as standard deviation of replicated analyses. Accuracy and precision were >2–5% for major elements and 5–10% for trace elements. The detection limit was 0.01% for the main oxides and 1–3 mg kg^−1^ for trace element concentrations, respectively.

The soil analyses were carried out at both the Laboratory of Plant Ecology (Department of Environmental and Prevention Sciences) and the Department of Physics and Earth Sciences of Ferrara University of Ferrara University. A 10 g subsample of dry soil was extracted in a 1:2.5 (vol/vol) aqueous solution and used to determine soil pH with a pH meter [Hanna Edge, Villafranca Padovana (PD), Italy]. A 500 mg subsample of dry soil, sieved with a 0.125 mm mesh, was used for analyzing total carbon (C) by a Shimadzu TOC-V_CSH_ (Shimadzu Corporation; Kyoto, Japan), connected with a solid sample module (Shimadzu SSM-5000A). A 200 mg subsample of dry soil, sieved with a 0.125 mm mesh, was extracted in 3 mL of selenous H_2_SO_4_ at 420 °C and analyzed for total nitrogen (N) concentration using the salicylate method, and for total phosphorus (P) concentration by the molybdenum blue method using a continuous flow autoanalyzer (FlowSys; Systea, Anagni, Italy). To determine total soil concentrations of K (as K_2_O), Ca (as CaO), Mg (as MgO), and Fe (as Fe_2_O_3_), a subsample of soil was sieved and subsequently pulverized using an agate mortar and subsequently embedded in a tablet for the X.R.F. analysis, as for the bedrock analyses.

### 4.3. Data Compilation and Statistical Analyses

The cumulative direct solar radiation (DSR) input at each plot was calculated by combining field-measured slope angle and aspect data according to Buffo et al. [63]. In the calculations, we assumed a linear trend of DSR values between slope increments. Then, we algebraically interpolated DSR values for intermediate slope angles at latitudes of 40° and 50° N. Further assuming a linear trend of latitudinal DSR values, we estimated DSR at latitude of 45° N by averaging the corresponding DSR values at latitudes of 40° and 50° N, respectively. Elevation was not included in the calculations due to the narrow elevation range of our plots.

A geochemical bedrock classification was obtained through cluster analysis of 32 geochemical variables using the Ward’s method based on the Euclidean distance. In order to avoid biases associated with the wide range of concentrations among the variables considered, the raw values were normalized to the maximum value using Formula (1):

X_normalized_ = X_i_/X_max_ (1)

where X_i_ is the rough value of a given variable for a plot site and X_max_ is the maximum rough value of a given variable across the whole set of plots.

The significance of differences among lithological clusters in terms of bedrock chemistry and soil chemistry was assessed by one-way ANOVAs and Dunn’s post hoc tests. The relationships between vegetation and bedrock chemistry were analyzed by Canonical Correspondence Analysis (CCA). To measure the association between species composition and lithological clusters, we used the Indicator Value (IndVal) index [64]. The IndVal index combines the average relative abundance and frequency of species occurrence in the lithological groups. We considered two classes of significance for the indicator species based on the *p* values associated to the IndVal: *p* < 0.05 and *p* < 0.01. The statistical analyses were performed using the software Past 4.13 [65].

## 5. Conclusions

We conclude that the vegetation investigated in our study was mostly made up of a set of species found more or less frequently found in silicicolous or calcicolous alpine plant communities in the Alps and other European mountains. Overall, there were no distinctive species uniquely characterizing grassland vegetation on serpentines s.l. or calc-schists s.l. Bedrock carbonate content was a major driver of variation in grassland vegetation, not necessarily related to soil pH. Detailed petrological analyses of bedrock revealed subtle differences in species composition associated with a complex interplay between protolith geochemistry, lithological genesis, and degree of metamorphism. Other factors additional to bedrock chemistry, in particular solar radiation and soil moisture content, also played a role in structuring the vegetation of alpine grasslands.

## Figures and Tables

**Table 1:** Mean (±1 SE) values of the geochemical variables in the six lithological groups, with the associated F values and *p* levels obtained by one-way ANOVAs. For each variable, the means followed by the same letter do not differ significantly (*p* < 0.05) based on Dunn’s post hoc tests. FS: moderately felsic schists; MO: meta-ophiolites; OC: meta-ophicalcites; CS: calc-schists s.s.; TS: talc-schists; SS: serpentine-schists.

Geochemical Variables	Lithological Group						ANOVAS’ Summary	
	FS (n = 4)	MO (n = 7)	OC (n = 4)	CS (n = 5)	TS (n = 3)	SS (n = 7)	F	*p*
SiO_2_ (%)	59.87 ± 1.48 a	54.96 ± 4.68 a	35.08 ± 2.46 bc	13.37 ± 1.37 c	43.16 ± 2.06 abc	47.58 ± 1.63 ab	27.82	<0.001
TiO_2_ (%)	0.79 ± 0.08 a	0.61 ± 0.03 ab	0.27 ± 0.06 bc	0.13 ± 0.02 c	0.11 ± 0.05 c	1.18 ± 0.16 a	18.73	<0.001
Al_2_O_3_ (%)	18.71 ± 1.23 a	15.49 ± 1.30 ab	6.67 ± 1.04 bc	2.93 ± 0.53 c	2.07 ± 0.11 c	14.51 ± 0.48 ab	45.15	<0.001
Fe_2_O_3_ (%)	6.25 ± 0.45 ab	5.10 ± 0.36 bc	2.63 ± 0.41 cd	1.97 ± 0.30 d	5.78 ± 1.17 abc	9.98 ± 1.13 a	15.61	<0.001
MnO (%)	0.08 ± 0.01 b	0.20 ± 0.04 a	0.08 ± 0.02 b	0.07 ± 0.01 b	0.09 ± 0.01 b	0.17 ± 0.02 a	5.53	<0.001
MgO (%)	3.38 ± 0.37 bcd	2.23 ± 0.23 cd	4.50 ± 0.76 abc	1.14 ± 0.13 d	33.32 ± 2.94 a	8.60 ± 0.55 ab	158.30	<0.001
CaO (%)	1.84 ± 0.80 c	8.22 ± 2.80 c	22.85 ± 1.58 ab	36.54 ± 1.56 a	6.82 ± 3.68 bc	9.93 ± 1.38 bc	32.83	<0.001
Na_2_O (%)	3.43 ± 0.23 a	1.10 ± 0.14 ab	0.67 ± 0.14 bc	0.39 ± 0.03 bc	0.14 ± 0.04 c	3.32 ± 0.29 a	47.85	<0.001
K_2_O (%)	2.44 ± 0.41 a	2.81 ± 0.31 a	1.44 ± 0.14 ab	0.36 ± 0.15 bc	0.00 ± 0.00 c	0.29 ± 0.13 bc	24.07	<0.001
P_2_O_5_% (%)	0.16 ± 0.01 a	0.08 ± 0.02 bc	0.08 ± 0.01 abcd	0.07 ± 0.01 cd	0.01 ± 0.00 d	0.15 ± 0.04 a	4.67	<0.001
L.O.I. (%)	3.06 ± 0.32 d	9.19 ± 2.15 bc	25.73 ± 0.75 ab	43.03 ± 3.16 a	8.52 ± 1.50 bcd	4.29 ± 1.12 cd	62.75	<0.001
Ba (mg kg^−1^)	834.25 ± 155.27 a	421.01 ± 51.50 a	279.15 ± 27.08 ab	82.94 ± 13.27 bc	18.43 ± 6.28 c	43.61 ± 13.59 c	24.59	<0.001
Ce (mg kg^−1^)	55.25 ± 11.85 a	29.64 ± 3.10 ab	9.83 ± 4.52 bc	4.74 ± 1.99 c	1.60 ± 0.83 c	3.31 ± 1.51 c	19.00	<0.001
Co (mg kg^−1^)	5.80 ± 2.04 d	17.87 ± 7.27 bc	13.93 ± 1.91 cd	9.02 ± 1.81 cd	96.43 ± 7.29 a	37.73 ± 5.91 ab	44.35	<0.001
Cr (mg kg^−1^)	59.53 ± 21.09 cd	136.76 ± 19.51 bc	167.25 ± 36.38 abc	22.48 ± 2.42 d	2718.10 ± 646.77 a	251.74 ± 35.34 ab	33.52	<0.001
Cu (mg kg^−1^)	17.95 ± 6.79 b	48.30 ± 9.87 a	20.40 ± 2.38 ab	17.44 ± 4.16 b	17.70 ± 8.16 ab	30.14 ± 7.33 ab	2.75	<0.001
Ga (mg kg^−1^)	19.25 ± 1.78 a	16.99 ± 2.02 a	6.73 ± 1.87 bc	2.88 ± 0.22 c	0.00 ± 0.00 c	11.44 ± 1.17 ab	19.86	<0.001
Hf (mg kg^−1^)	5.23 ± 0.23 a	3.47 ± 0.54 ab	1.20 ± 0.17 bc	0.24 ± 0.07 c	0.00 ± 0.00 c	1.97 ± 0.38 b	21.22	<0.001
La (mg kg^−1^)	11.63 ± 5.22 a	7.93 ± 3.69 ab	0.00 ± 0.00 b	3.54 ± 3.54 ab	0.00 ± 0.00 b	3.99 ± 1.17 ab	1.70	0.17
Nb (mg kg^−1^)	14.40 ± 1.05 ab	15.34 ± 1.56 a	3.38 ± 0.82 c	0.86 ± 0.50 c	0.30 ± 0.12 c	4.64 ± 0.56 bc	37.98	<0.001
Nd (mg kg^−1^)	33.13 ± 9.06 a	21.27 ± 2.21 a	6.68 ± 1.34 b	6.46 ± 1.10 b	0.00 ± 0.00 b	7.51 ± 1.07 b	11.94	<0.001
Ni (mg kg^−1^)	23.75 ± 7.68 bc	91.53 ± 17.56 a	81.23 ± 9.64 ab	20.96 ± 1.64 c	1653.33 ± 150.76 a	84.83 ± 5.86 a	214.90	<0.001
Pb (mg kg^−1^)	7.43 ± 1.61 abc	14.61 ± 1.96 a	11.43 ± 2.13 a	10.64 ± 2.84 ab	3.83 ± 0.86 bc	3.54 ± 0.55 c	6.27	<0.001
Rb (mg kg^−1^)	61.20 ± 11.56 ab	104.01 ± 12.06 a	47.25 ± 6.13 ab	19.38 ± 4.28 bc	1.47 ± 0.09 c	5.36 ± 1.90 c	25.05	<0.001
Sc (mg kg^−1^)	10.70 ± 1.39 ab	10.37 ± 2.66 ab	1.43 ± 0.56 bc	0.10 ± 0.03 c	16.73 ± 5.59 ab	18.39 ± 1.62 a	10.92	<0.001
Sr (mg kg^−1^)	129.75 ± 48.68 bc	207.73 ± 39.00 b	223.93 ± 19.10 ab	629.04 ± 97.89 a	9.23 ± 5.56 c	201.47 ± 19.82 b	15.45	<0.001
Th (mg kg^−1^)	7.85 ± 1.04 a	7.00 ± 0.70 a	2.38 ± 0.27 ab	1.14 ± 0.33 bc	0.17 ± 0.09 c	0.40 ± 0.11 bc	37.50	<0.001
V (mg kg^−1^)	135.93 ± 35.66 ab	115.76 ± 12.84 ab	60.23 ± 11.88 bc	29.52 ± 2.46 c	66.57 ± 4.89 bc	188.43 ± 11.56 a	15.14	<0.001
Y (mg kg^−1^)	29.03 ± 5.22 a	23.56 ± 2.19 ab	12.08 ± 2.01 bc	10.58 ± 2.72 bc	1.83 ± 0.49 c	32.37 ± 3.46 a	12.37	<0.001
Zn (mg kg^−1^)	73.18 ± 7.25 ab	91.83 ± 7.59 a	41.10 ± 5.58 bc	19.02 ± 2.03 c	70.03 ± 11.11 ab	72.57 ± 7.61 ab	13.36	<0.001
Zr (mg kg^−1^)	230.58 ± 5.79 a	173.84 ± 17.34 ab	68.00 ± 5.03 bc	18.66 ± 2.31 c	1.43 ± 1.24 c	128.27 ± 24.08 ab	21.94	<0.001
CaCO_3_ (%)	0.00 ± 0.00 c	9.14 ± 4.11 bc	34.00 ± 2.83 ab	71.20 ± 1.80 a	0.00 ± 0.00 c	1.86 ± 1.86 c	93.63	<0.001

**Table 2:** Mean cover, visually estimated according to a 1–10 categorical scale, of 76 vascular plant species sorted by lithological groups (FS: relatively felsic schists; MO: meta-ophiolites; OC: meta-ophicalcites; CS: calc-schists s.s.; TS: talc-schists; SS: serpentine schists) in the 30 plots. For each lithological group the indicator species, assessed by the *p* values associated to the Indval index, are highlighted within bordered boxes (* *p* < 0.05; ** *p* < 0.01). The first column contains abbreviations indicating the syntaxonomic rank of each species: AC: *Arabidetalia caeruleae*; CC: *Caricetalia curvulae*; DH: *Drabetalia hoppeanae*; OE: *Oxytropido-Elynetalia*; SC: *Seslerietalia caeruleae*; SH: *Salicetalia herbaceae*; Ot.: other syntaxa. The third column contains the species abbreviations used in Figure 4. Rare species, occurring in <3 plots, are listed in Supplementary Table S3.

		FS				MO							OC				CS					TS			SS						
Plot		1	2	3	4	5	6	7	8	9	10	11	12	13	14	15	16	17	18	19	20	21	22	23	24	25	26	27	28	29	30
*Indicator species*																															
*FS*																															
*SC—Bartsia alpina* (*)	Bar alp		1		1	1	1		1			1																			1
*Ot.—Mutellina adonidifolia* (*)	Mut ado		1		1		1					1					1														
*CC—Phyteuma hemisphaericum* (*)	Phy hem	1		1				1											1						1			1			
*CC – Scorzoneroides helvetica* (*)	Sco hel	1		1				1									1									1	1	1			
*CC—Veronica bellidioides* (*)	Ver bel	1		1			1										1								1				1		1
*MO*																															
*DH—Achillea nana* (**)	Ach nan					1		1		1		1																			
*CC—Pedicularis kerneri* (**)	Ped ker		1			1	1		1			1															1	1			
*AC—Salix retusa* (**)	Sal rtu					1		2	4	1											1										
*Ot.—Anthoxanthum nipponicum* (*)	Ant nip							1		1		1	1																		
*SC—Festuca nigricans* (*)	Fes nig							2	2	3		2			2							1	1				2			2	
*Ot.—Bistorta vivipara* (*)	Bis viv		1		1	1	1	1	1	1	1	2	1		2	1		1		1	1			1				1			1
*OC*																															
*SC—Helianthemum nummularium* subsp. *grandiflorum* (**)	Hel num													1	1	1								1							
*CC—Silene acaulis* (*)	Sil aca					1	1		1			1	1		1	1		1						1							
*CS*																															
*SC—Helianthemum oelandicum* subsp. *alpestre* (**)	Hel oel		1		1		1				1		1				1	1	2	1	1										
*CC—Botrychium lunaria* (*)	Bot lun																1			1	1	1		1			1				
*SC—Draba aizoides* subsp. *aizoides* (*)	Dra aiz					1													1	1											
*OE—Erigeron uniflorus* (*)	Eri uni					1						1	1							1	1										
*SC—Pedicularis rosea* subsp. *allionii* (*)	Ped ros																		1	1								1			1
*CC—Phyteuma globulariifolium* subsp. *pedemontanum* (*)	Phy glo						1					1								1	1										
*Ot.—Plantago alpina* (*)	Pla alp					1											1	1											1		
*Ot.—Poa alpina* (*)	Poa alp					1	1		1	1		1	1		1			1	1	1	1			1	1				1		1
*DH—Saxifraga oppositifolia* subsp. *oppositifolia* (*)	Sax opp								1				1						1		1										
*CC—Jacobaea incana* (*)	Jac inc										1							1			1				1				1		
*TS*																															
*CC—Luzula lutea* subsp. *lutea* (**)	Luz lut							1	1	1												1		1	1						
*SC—Potentilla crantzii* subsp. *crantzii* (**)	Pot cra																					1	2	2	1		1				
*Ot.—Thymus praecox* subsp. *polytrichus* (**)	Thy pra							1						1								1	1	1						1	
*CC—Agrostis rupestris* subsp. *rupestris* (*)	Agr rup		1		1		1	1			1	1	1	1	1		1	1			1	2	2	1		1	3	1		2	
*CC—Dianthus furcatus* (*)	Dia fur														1			1					1	1							
*Ot.—Festuca rubra* (*)	Fes rub														1							1		1							
*SC—Galium anisophyllon* (*)	Gal ani																1					1		1							
*CC—Oreojuncus trifidus* (*)	Ore tri	2						2	1	1					1							4	1		1	1				1	
*SS*																															
*CC—Juncus jacquinii* (**)	Jun jac																											1	1	1	1
*Ot.—Alchemilla vulgaris* (*)	Alc vul									1																	1	1			
*CC—Festuca halleri* (*)	Fes hal																			1					1						1
*CC—Festuca scabriculmis* subsp. *luedii* (*)	Fes lue	4																								2	2	1			
*CC—Nardus stricta* (*)	Nar str																							3		2		2		2	
*Ot.—Pulsatilla alpina* subsp. *apiifolia* (*)	Pul alp							1																				1	1		
*CC—Sempervivum montanum* (*)	Sem mon					1	1	1									1								1				1		1
*Other species*																															
*OE—Carex myosuroides*	Car myo		2		3	1	3				2		1	1	1	2	1	2	2	1								1	1		2
*Ot.—Campanula scheuchzeri*	Cam sch		1		1			1		1	1		1		1					1	1	1	1	1			1	1		1	
*Ot.—Carex sempervirens* subsp. *sempervirens*	Car sem	1	3		2			1			2	1		1	3			1	1				2	2		2	1			1	
*Ot.—Lotus corniculatus* subsp. *alpinus*	Lot cor							1		2	2						1	2		1		2			1		1	1		1	
*CC—Carex curvula* subsp. *curvula*	Car cur			3								1	1				1		2	2					1	1			1		1
*CC—Euphrasia minima*	Eup min		1		1	1	1		1				1						1		1				1	1					
*CC—Helictochloa versicolor* subsp. *versicolor*	Hel ver			2	1		2				1					1					1				1	1		3			1
*CC—Gentianella ramosa*	Gen ram	1		1		1									1		1									1	1	1	1		
*SC—Sesleria caerulea*	Ses cae		1		2	1					2			2		1		2	1	2											
*CC—Soldanella alpina* subsp. *alpina*	Sol alp		1		1			1		1			1					1		1				1			1				
*Ot.—Pilosella officinarum*	Pil off	1									1				1			1		1			1					2		1	
*CC—Trifolium alpinum*	Tri alp	2		2																			2	2		2	1		3	1	
*SC—Anthyllis vulneraria* subsp. *alpicola*	Ant vul		1		1	1	1	1										1					1								
*SC—Myosotis alpestris*	Myo alp									1			1		1					1				1				1	1		
*SC—Oxytropis montana*	Oxy mon		1		1	1	1									1		1		1											
*AC—Carex parviflora*	Car par								1	1		1	1					1	1												
*OE—Dryas octopetala* subsp. *octopetala*	Dry oct		4								1					7		1	1	1											
*SC—Festuca violacea*	Fes vio				1	1	1						2				3								3						
*CC—Minuartia recurva*	Min rec	1		1													1				1		1		1						
*OE –Salix serpillifolia*	Sal ser					1	1						2							1	1										3
*SC—Festuca pumila*	Fes pum										1			1		1		1			3										
*SC—Gentiana verna*	Gen ver				1		1												1											1	1
*CC—Geum montanum*	Geu mon							1		1														1				1	3		
*Ot.—Sempervivum arachnoideum*	Sem ara	1												1			1		1											1	
*SC—Anemonoides baldensis*	Ane bal					2				1								1		1											
*OE—Arenaria ciliata*	Are cil					1	1										1								1						
*SC—Aster alpinus* subsp. *alpinus*	Ast alp		1		1									1		1															
*CC—Hieracium alpinum*	Hie alp	2						1									1								1						
*Ot.—Achillea erba-rotta* subsp. *erba-rotta*	Ach erb												1								1				1						
*CC—Armeria alpina*	Arm alp											1												1	1						
*SH—Omalotheca supina*	Oma sup			1															1	1											
*Ot.—Leucanthemum coronopifolium*	Leu cor							1		1												1									
*CC—Cherleria sedoides*	Che sed			1															1												1
*SC—Sabulina verna*	Sab ver													1						1					1						
*CC—Potentilla aurea* subsp. *aurea*	Pot aur			1																								2	1		
*SC—Saxifraga paniculata*	Sax pan									2				1							1										
*SC—Trifolium thalii*	Tri tha																					1						1		3	
*CC—Veronica allionii*	Ver all														2							1								1	

## Data Availability

The data presented in this study are available upon request from the corresponding author. The data are not publicly available due to privacy.

## Supplementary Materials

The following supporting information can be downloaded at: https://www.mdpi.com/article/10.3390/plants13162280/s1, Table S1: Location of the plots with description of the lithotypes as listed in the Geological maps, Table S2: Synthetic petrological overview of the six lithological groups, Table S3: List of the rare species.
