# Supplementary material for: Fine-Scale Lithogeochemical Features Influence Plant Distribution Patterns in Alpine Grasslands in the Western Alps of Italy
Source: Plants (Basel). 2024 Aug 16;13(16):2280. doi: 10.3390/plants13162280 (PMC11360244; doi:10.3390/plants13162280)
Supplement: Supplementary file 1 [file plants-13-02280-s001.zip › plants-3067003-supplementary.pdf]

Supplementary Table S1 – Location of the plots with description of the lithotypes as listed in the Geological maps.

**Plots** (Chronological order)	**Plots** (Dendrogram order)	**Location**	**Lithotypes described in geological maps**
1, 2, 3, 4	29, 22, 21, 23	***Colle del Sabbione***<br>Municipality: Bussoleno<br>Province: Turin<br>Nation: Italy<br><br>45° 3'43.19"N<br>7° 7'58.87"E	**ρ**: Prasinites, simple and granatipher amphibiolites, prasinites with ocellar structure mostly enriched in clorite (ovardites), various glaucophane rocks (p.p. gastaldite) often grenatiferous; eclogytes; chloromelanites and jadeitite<br>**σ**: serpentine and serpentine-schists
5, 6, 7	14, 13, 15	***Colle della Vecchia***<br>Municipality: Usseaux<br>Province: Turin<br>Nation: Italy<br><br>45° 4'23.27"N<br>7° 1'32.89"E	**ρ**: Prasinites, simple and granatipher amphibiolites, prasinites with ocellar structure and mostly enriched in clorite (ovardites), various glaucophane rocks (p.p. gastaldite) often grenatiferous; eclogytes; chloromelanites and jadeitites<br>**cs**: calc-schists and phyllite, sometimes with carbonaceous or graphitic pigment, often chloritoid (sismondine), with crystalline limestone banks and lenses, and small intercalations of minute gneiss and mica-schists
8, 9, 10, 11	10, 17, 19, 18	**Plateau below *Pointe du Lamet***<br>Municipality: Val-Cenis<br>Province: Lanslebourg-Mont-Cenis<br>Nation: France<br><br>45°14'41.84"N<br>6°58'26.58"E	Calcareous Bündner schists poor in ophiolites<br>Flysch-type Bündner schists
12, 13, 14, 15	7, 9, 8, 11	**Plateau around *Rocca Turo***<br>Municipality: Balme<br>Province: Turin<br>Nation: Italy<br><br>45°17'56.77"N<br>7° 8'43.64"E	**ρ**: Prasinites, simple and granatipher amphibiolites, prasinites with ocellar structure mostly enriched in clorite (ovardites), various glaucophane rocks (p.p. gastaldite) often grenatiferous; eclogytes; chloromelanites and jadeitite<br>**cs**: calc-schists and phyllite, sometimes with carbonaceous or graphitic pigment, often chloritoid (sismondine), with crystalline limestone banks and lenses, and small intercalations of minute gneiss and mica-schists
16, 17, 18, 19	12, 1, 20, 24	**SE of *Lago Corona* near *Rio Lussert***<br>Municipality: Cogne<br>Province : Aosta<br>Nation: Italy<br><br>45°38'50.57"N<br>7°25'3.76"E	**EML**: Undifferentiated metamorphic base. Complex of eclogitic mica-schists, derived from paraschists and minor granitoids, with local pre-Alpine granulitic relicts, abundant eclogites, scarce marbles and retrocession into incipient to very pervasive green schist facies<br>**EMLb**: Albitic minute gneiss with local eclogitic relicts. Lithotypes with strong retrocession in green-schist facies, micaceous-chlorite epidote, greenish biotite, porphyroblastic albite and metastable relicts of the original eclogitic association.
20, 21, 22, 23, 24	5, 16, 6, 4, 2	**Between *Dora di Valgrisanche* and *Pian di Vaudet* near *Rifugio Bezzi***<br>Valgrisenche<br>Province Aosta<br>Nation: Italy<br><br>45°31'59.86"N<br>7° 1'30.02"E	**t_2**: Saccharoid marble limestones; micaceous grey limestones, black limestones with traces of fossils (crinoids?), white limestones in slabs, dolomite and carniole; calc-schists<br>**cs**: Calc-schists and phyllodes with banks and lenses of crystalline and cipolin limestones with minor intercalations of minute gneiss and mica-schists
25, 26, 27	25, 26, 27	**Between *Lago Gabiet*'s Northern and Southern dams**<br>Municipality: Gressoney-La-Trinité<br>Province: Aosta<br>Nation: Aosta, Italy<br><br>45°51'55.37"N<br>7°51'7.66"E	**cs**: Calc-schists and phyllodes with banks and lenses of crystalline limestones and minor intercalations of minute gneiss, mica-schists and quartzites<br>**ρ**: Prasinites, simple and grenadiferous amphibolites; Miscellaneous glaucophanic rocks<br>**σ**: Serpentines and serpentine-schists; talc-schists and actinolytic chlorite-schists, sometimes with tourmaline; grenadiferous chlorite-schists (molasses): garnets, pyroxenites and epidosites
28, 29, 30	3, 30, 28	***Colle del Nivolet***<br>Valsavarenche<br>Province: Aosta<br>Nation: Italy<br><br>45°28'44.11"N<br>7° 8'38.89"E	**ρ**: Prasinites, simple and granatipher amphibiolites, prasinites with ocellar structure mostly enriched in clorite (ovardites), various glaucophane rocks (p.p. gastaldite) often grenatiferous; eclogytes; chloromelanites and jadeitites<br>**cs**: Calc-schists and phyllite, sometimes with carbonaceous or graphitic pigment, often chloritoid (sismondine), with crystalline limestone banks and lenses, and small intercalations of minute gneiss and mica-schists<br>**gs_1**: Tabular gneisses, minute gneisses sometimes with graphite pigment, various mica schists with white mica, amphibolic gneisses associated with 'ghiandone' gneiss (p.p. Carboniferous with Piedmont facies?)

Supplementary Table S2 – Synthetic petrological overview of the six lithological groups with detailed description of one representative sample for each group.

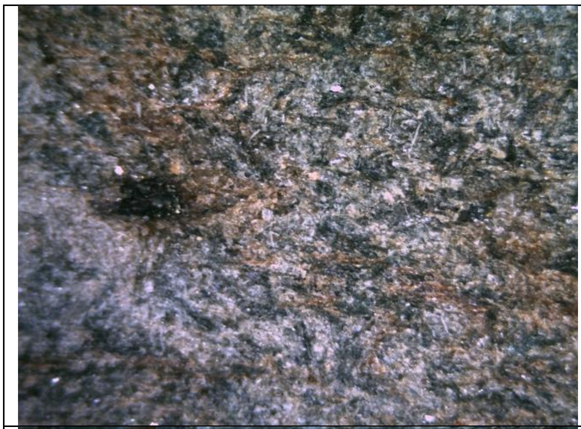	Plot number: 1<br>Location: ***Lago Corona***<br>Classification (binary): Calc-schist s.l.<br>Lithological group: Relatively Felsic schist (FS)<br>Petrological classification: Mica-schist<br>Protolith: Sialic rock rich in feldspar, muscovite and iron hydroxides<br>Metamorphic facies: Green-schist facies leaning to zeolites<br>Soil pH: 5.73
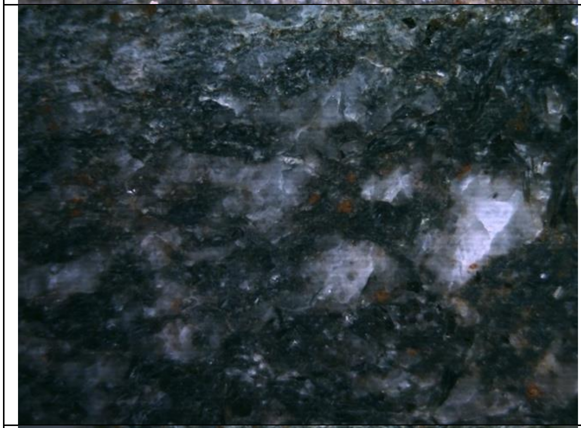	Plot number: 7<br>Location: ***Rocca Turo***<br>Classification (binary): Ophiolite<br>Lithological group: Meta-ophiolite (MO)<br>Petrological classification: Actinolitic chlorite-schist<br>Protolith: Femic-sialic rock of pelitic origin with feldspar, muscovite quartz, amphiboles and secondary calcite<br>Metamorphic facies: Green-schist facies<br>Soil pH: 6.10
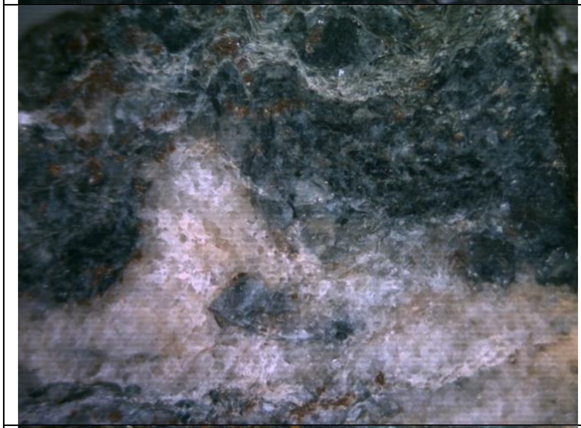	Plot number: 12<br>Location: ***Lago Coronas***<br>Classification (binary): Calc-schist s.l.<br>Lithological group: Meta-ophicalcite (OC)<br>Protolith: femic-Sialic rich in quartz, feldspar, iron hydroxydes, chlorite, talc, serpentine and amphiboles alongside with calcite<br>Metamorphic facies: Green-schist facies leaning towards amphiboles<br>Soil pH: 5.74
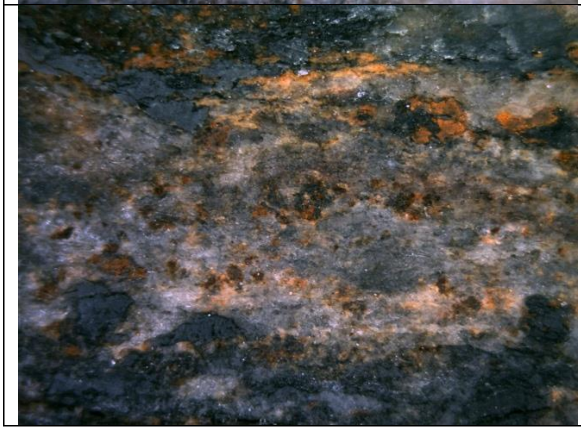	Plot number: 19<br>Location: ***Lago Coronas***<br>Classification (binary): Calc-schist s.l.<br>Lithological group: Calc-schist s.s. (CS)<br>Protolith: Magmatic alkaline rock rich in quartz, feldspar, iron hydroxydes, chlorite, talc, serpentine, graphite, pyrossen, micas and amphiboles alongside with abundant calcite<br>Metamorphic facies: Green-schist facies<br>Soil pH: 6.18

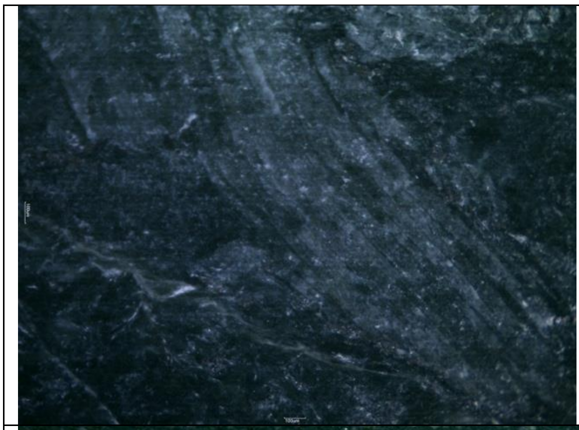	Plot number: 21<br>Location: Colle del Sabbione<br>Classification (binary): Ophiolite<br>Lithological group: Talc-schist (TS)<br>Protolith: Ultrafemic rock, rich in chlorite and serpentine. Talc striations are visible<br>Metamorphic facies: Green-schist facies<br>Soil pH: 5.82
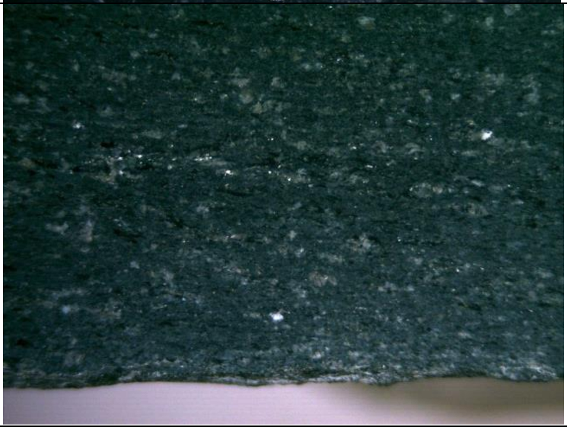	Plot number:<br>Location: ***Lake Gabiet***<br>Classification (binary): Ophiolite<br>Lithological group: Serpentine-schist (SS)<br>Protolith: Femic rock, rich in amphibole, chlorite and serpentine. Minor traces of feldspar<br>Metamorphic facies: Green-schist facies<br>Soil pH: 5.60

Supplementary Table S3 – List of the rare species, recorded in < 3 plots.

	FS				MO							OC				CS					TS			SS						
plot	1	2	3	4	5	6	7	8	9	10	11	12	13	14	15	16	17	18	19	20	21	22	23	24	25	26	27	28	29	30
Alchemilla pentaphyllea			1																									1		
Alopecurus alpinus																												1		
Alyssum montanum										1									1											
Androsace adfinis																								1						
Antennaria dioica										1												4								
Astragalus alpinus									1																					
Bellidiastrum michelii					1											1														
Biscutella laevigata									1																					
Bupleurum ranunculoides			1																							1				
Carex capillaris subsp. *capillaris*		1																												
Carex foetida																												1		
Carlina acaulis									1																					
Cerastium arvense																1														
Cerastium arvense subsp. *strictum*																					1									
Coeloglossum viride								1																						
Dianthus barbatus																					1								2	
Facchinia rupestris																	1		1											
Festuca scabriculmis																					2								2	
Gentiana acaulis																										1	1			
Gentiana brachyphylla																				1				1						
Gentiana nivalis					1											1														
Hieracium armerioides							1									1														
Homogyne alpina							1										1													

Leontodon hispidus							1						1								
Leontodon hispidus subsp. *hispidus*															1						
Leontopodium alpinum											1	1									
Leucanthemopsis alpina																		1			
Luzula alpinopilosa subsp. *alpinopilosa*									1												
Luzula spicata																				1	
Luzula sudetica										1											
Cherleria capillacea														1							
Nigritella nigra							1														
Oxytropis campestris				1										1							
Oxytropis helvetica											2										
Oxytropis lapponica									4												
Parnassia palustris subsp. *palustris*																			1		
Paronychia polygonifolia																				1	
Plantago atrata subsp. *atrata*													1								
Potentilla grandiflora																					1
Primula latifolia subsp. *graveolens*						1															
Pulsatilla vernalis								1													
Ranunculus gr. *montanus*	1																1				
Salix herbacea									2	2											
Salix reticulata						1															
Saxifraga exarata																1					
Saxifraga retusa										1											
Scabiosa lucida subsp. *lucida*			1	1																	
Sedum atratum															1	1					
Selaginella selaginoides					1																
Sibbaldia procumbens		1																			
Taraxacum oreophilum										1											

Thesium alpinum									1	1		
Noccaea sylvia								1	1			
Trifolium badium		1										
Trifolium pratense subsp. *nivale*							2				1	
Koeleria spicata subsp. *ovatipaniculata*						1						
Vaccinium uliginosum subsp. *microphyllum*			1									1
Valeriana celtica	1											1
Veronica alpina				1								
Veronica aphylla				1								
Veronica fruticans					1							
